# Supplementary material for: Testing the function of dromaeosaurid (Dinosauria, Theropoda) ‘sickle claws’ through musculoskeletal modelling and optimization
Source: PeerJ. 2019 Aug 28;7:e7577. doi: 10.7717/peerj.7577 (PMC6717499; doi:10.7717/peerj.7577)
Supplement: Figures S1–S5 [file peerj-07-7577-s001.doc]

Supplementary Material for

**Testing the function of dromaeosaurid (Dinosauria, Theropoda) ‘sickle claws’ through musculoskeletal modelling and optimization**

P.J. Bishop^1,2^

^1^ Structure and Motion Laboratory, Comparative Biomedical Sciences, Royal Veterinary College, Hatfield, United Kingdom.

^2^Geosciences Program, Queensland Museum, Brisbane, Australia.

Corresponding email: [pbishop@rvc.ac.uk](mailto:pbishop@rvc.ac.uk).


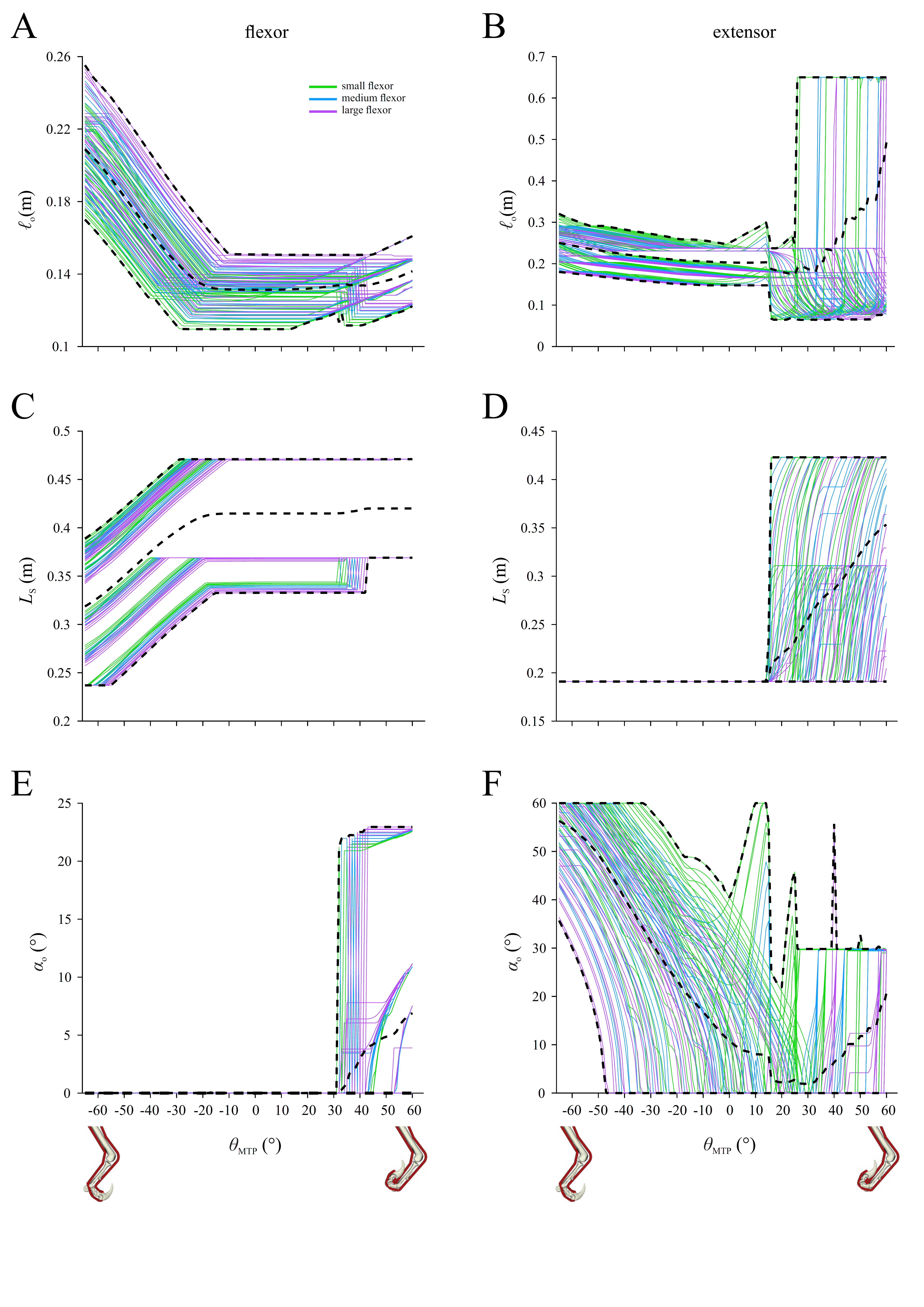
**Supplementary figures**

**Figure S1.** Optimal values of musculotendon parameters for the initial system configuration, plotted against metatarsophalangeal joint angle (*θ*_MTP_) and parsed by flexor muscle volume. (A, B) Optimal fibre length. (C, D) Tendon slack length. (E, F) Pennation angle. Panels A, C and E are for the flexor muscle; panels B, D and F are for the extensor muscle. Black dashed lines denote minimum, maximum and mean curves across all combinations.


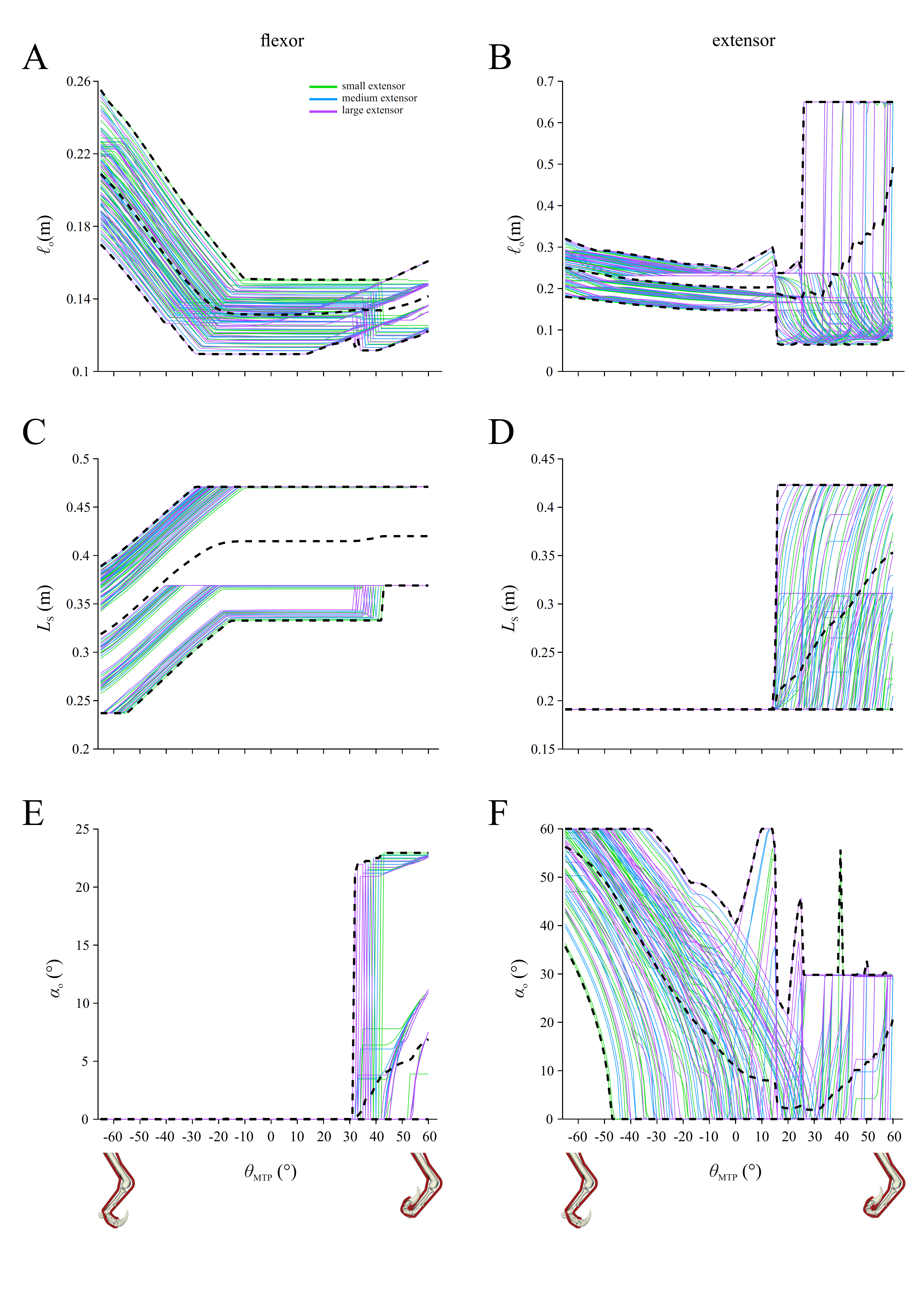


**Figure S2.** Optimal values of musculotendon parameters for the initial system configuration, plotted against metatarsophalangeal joint angle (*θ*_MTP_) and parsed by extensor muscle volume. (A, B) Optimal fibre length. (C, D) Tendon slack length. (E, F) Pennation angle. Panels A, C and E are for the flexor muscle; panels B, D and F are for the extensor muscle. Black dashed lines denote minimum, maximum and mean curves across all combinations.


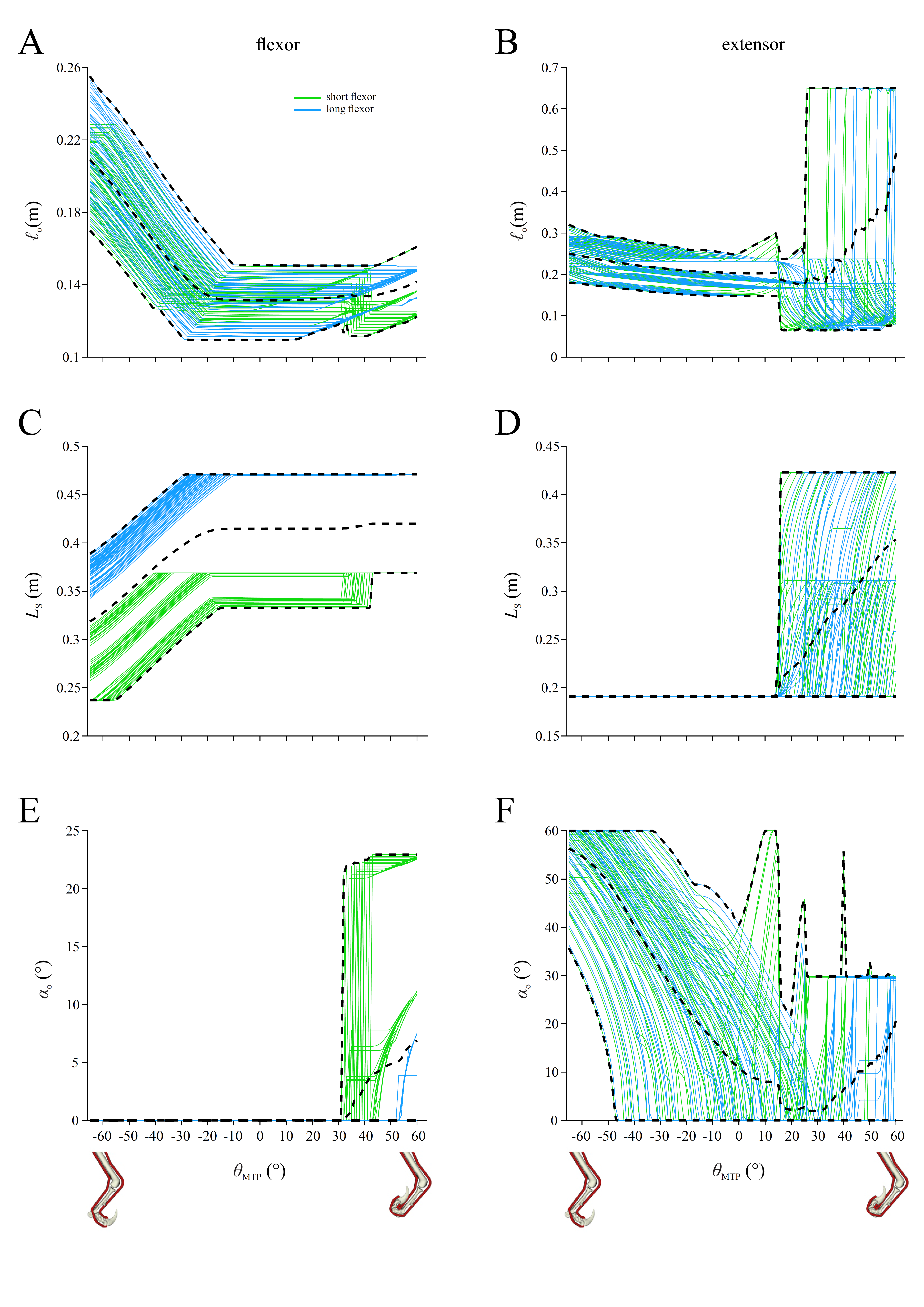


**Figure S3.** Optimal values of musculotendon parameters for the initial system configuration, plotted against metatarsophalangeal joint angle (*θ*_MTP_) and parsed by flexor muscle length. (A, B) Optimal fibre length. (C, D) Tendon slack length. (E, F) Pennation angle. Panels A, C and E are for the flexor muscle; panels B, D and F are for the extensor muscle. Black dashed lines denote minimum, maximum and mean curves across all combinations.


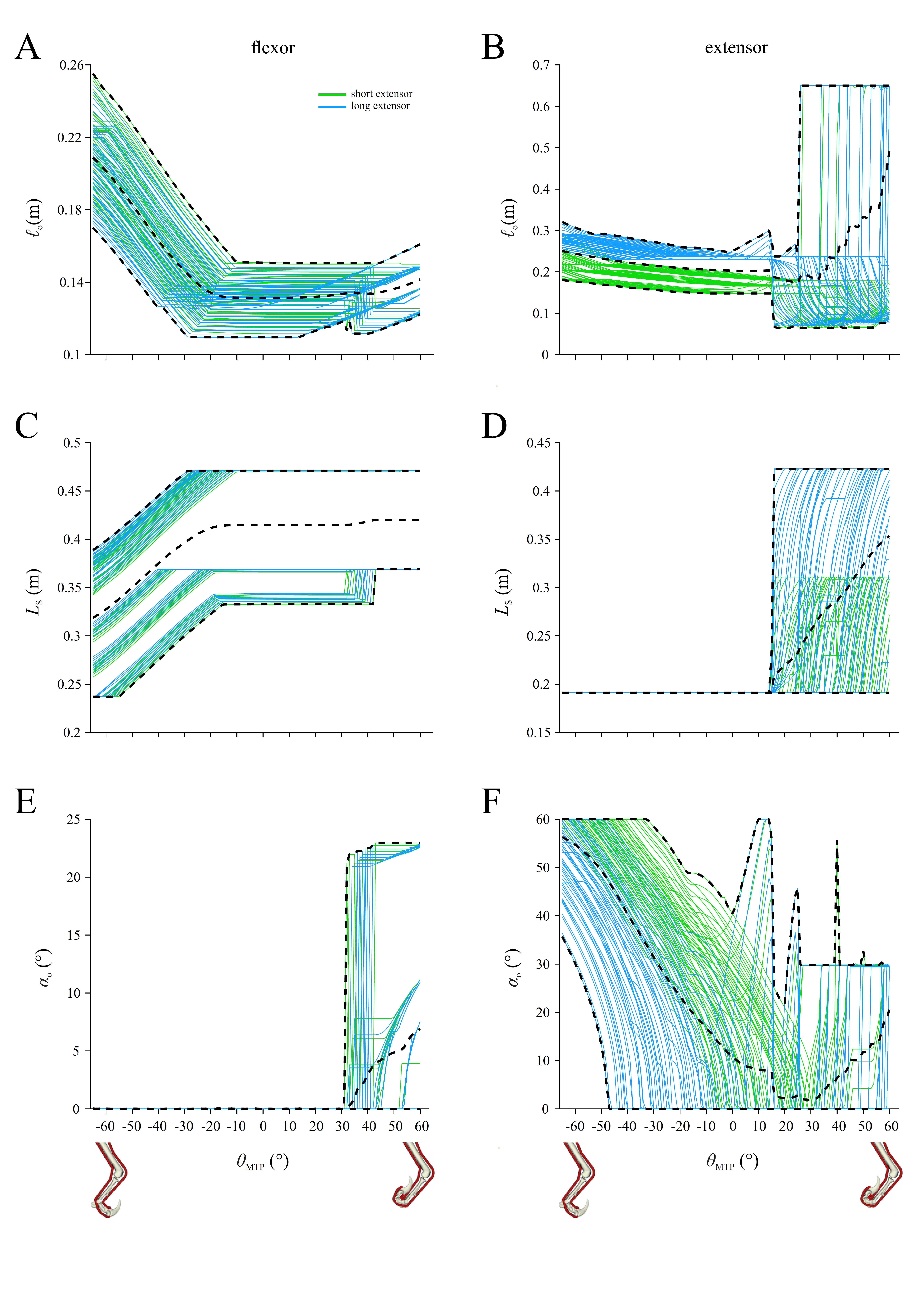


**Figure S4.** Optimal values of musculotendon parameters for the initial system configuration, plotted against metatarsophalangeal joint angle (*θ*_MTP_) and parsed by extensor muscle length. (A, B) Optimal fibre length. (C, D) Tendon slack length. (E, F) Pennation angle. Panels A, C and E are for the flexor muscle; panels B, D and F are for the extensor muscle. Black dashed lines denote minimum, maximum and mean curves across all combinations.


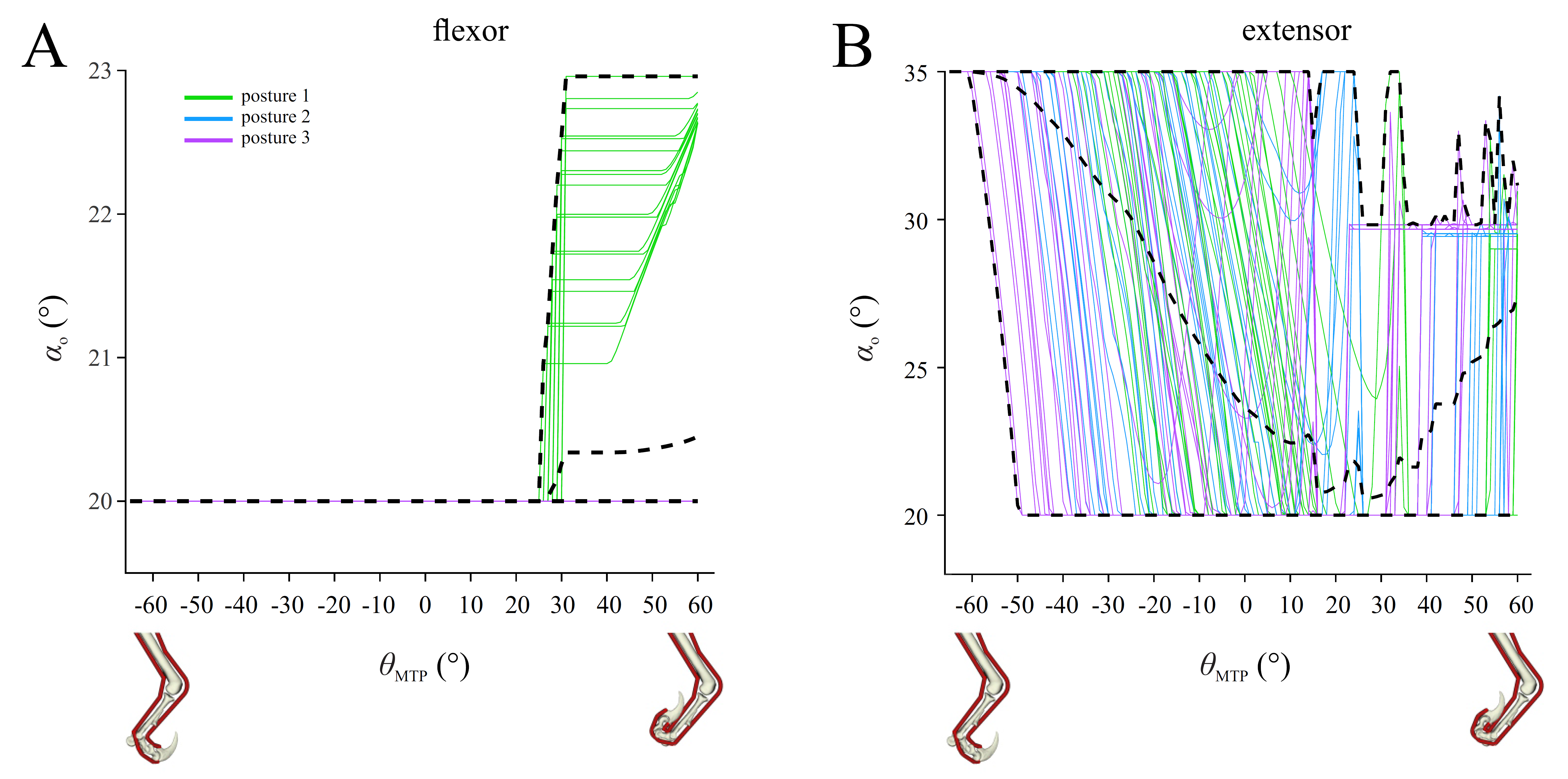


**Figure S5.** Optimal values for flexor muscle (A) and extensor muscle (B) pennation angle in the optimization with more restricted bounds on pennation angle, plotted against metatarsophalangeal joint angle (*θ*_MTP_). Results are parsed by posture. Black dashed lines denote minimum, maximum and mean curves across all combinations. Note that for both muscles, optimal pennation angle tends towards the upper or lower bounds of allowable values across at least part (sometimes all) of the range of *θ*_MTP_, especially for postures 2 and 3.
