# Supplementary material for: Testing the function of dromaeosaurid (Dinosauria, Theropoda) ‘sickle claws’ through musculoskeletal modelling and optimization
Source: PeerJ. 2019 Aug 28;7:e7577. doi: 10.7717/peerj.7577 (PMC6717499; doi:10.7717/peerj.7577)
Supplement: File S1 — See the included README document for an overview of the files. [file peerj-07-7577-s002.zip › Bishop, Supplementary Code and Files/README.doc]

This folder contains the OpenSim model files and MATLAB code that accompany the article “Testing the function of dromaeosaurid (Dinosauria, Theropoda) ‘sickle claws’ through musculoskeletal modelling and optimization” by P.J. Bishop (*PeerJ*, 2019).

The OpenSim model (.osim, and associated .vtp geometry files) is able to be viewed and modified using the open-source software OpenSim (<https://simtk.org/projects/opensim>). The MATLAB code (.m and .csv files) is able to be viewed using standard text editing software, in addition to MATLAB itself. Note that to run the code first requires installing the CasADi interface for MATLAB (<https://web.casadi.org/>), which is free to use. The MainScript.m file will run the optimization for a single combination, where preferences can be set in the Settings section. The MainScript_Batch.m file will run the optimization for all combinations, and again preferences can be set in the Settings section. Both main files also have basic functionality to plot the results obtained.

In the OpenSim model, the two interphalangeal (IP) joints are kinematically uncoupled from the metatarsophalangeal (MTP) joint so the user can explore each joint’s mobility independent of that of the others. The kinematic coupling used in the study is defined by Equations 1–2 in the text.
